# The influence of *CYP1A1* and *CYP1A2* polymorphisms on stroke risk in the Chinese population

**DOI:** 10.1186/s12944-020-01370-z

**Published:** 2020-10-12

**Authors:** Yan Mao, Lin Yang, Qian Chen, Guoqing Li, Yao Sun, Jiamin Wu, Zichao Xiong, Yuanwei Liu, Haiyue Li, Jianfeng Liu, Yong Zhang

**Affiliations:** 1 Department of Geriatrics, Xi’an Hospital of Traditional Chinese Medicine, Xi’an, 710021 Shaanxi China; 2 Department of Encephalopathy, Xi’an Hospital of Traditional Chinese Medicine, Xi’an, 710021 Shaanxi China; 3 Key Laboratory of Resource Biology and Biotechnology in Western China, Ministry of Education, School of Medicine, Northwest University, Xi’an, 710069 Shaanxi China; 4 The Second Department of Encephalopathy, Baoji Hospital of Traditional Chinese Medicine, Baoji, 721001 Shaanxi China

**Keywords:** Population study, Chinese, Stroke, *CYP1A1*, *CYP1A2*, Polymorphisms

## Abstract

**Backgrounds:**

Stroke is a sudden disorder of cerebral blood circulation. Many studies have illustrated that dyslipidemia, hypertension, diabetes, smoking and excessive drinking are the traditional risk factors for stroke. This study aimed to observe the relationship between *CYP1A1* and *CYP1A2* variants and stroke risk in the Chinese population.

**Methods:**

Agena MassARRAY Assay was used to genotype four single nucleotide polymorphisms (SNPs) in 477 cases and 480 controls. The chi-square test and logistic-regression analysis were used to explore the relationship between *CYP1A1* and *CYP1A2* variants and stroke risk.

**Results:**

Individuals with *CYP1A2* rs762551 C was associated with a lower risk of stroke than that of allele A. Age stratification analysis showed that rs762551 was only observed to be associated with a lower risk of stroke in ≤64ys age group. After gender stratification analysis, a significant association between rs762551 and stroke risk was found in males, but not in females. The four SNPs were found to be correlated with stroke risk in patients with hypertension, coronary heart disease, cerebral infarction and lacunar infarction.

**Conclusion:**

In this study, the results first showed that *CYP1A1* and *CYP1A2* variants were associated with stroke risk. Larger and well-designed studies are needed to confirm the results.

## Background

Generally speaking, stroke is a sudden disorder of cerebral blood circulation in the brain that occurs when blood clot blocks arteries transmitting blood from heart to brain [1]. At present, it ranks as the second leading cause of death in the world and the main cause of adult disability and death in most developing and developed countries [2]. According to the World Health Organization report in 2019, 5.5 million people died of stroke [3]. In China, there are over 2 million stroke patients and 6–7 million stroke survivors every year [4]. About 80% of stroke patients are ischemic [5, 6]. Many studies illustrated that dyslipidemia, hypertension, diabetes, smoking and excessive drinking are the traditional risk factors for stroke [7]. Moreover, genetic factors are also involved in stroke risk assessment.

Cytochrome P450s (CYPs) is a widely distributed oxyhemoglobin superfamily, whose main function is to activate the molecular oxygen of lipophilic organic compounds, including drugs, steroids, fatty acids, bioamines, prostaglandins, plant metabolites, estradiol, estrone, etc. [8]. Some of these compounds are carcinogens, but most of them are activated by phase I enzymes encoded by the CYP superfamily and transformed into active carcinogens [9]. The active carcinogen combine with DNA to form DNA adducts causing mutation and carcinogenesis. Epidemiological and basic scientific research have shown that *CYP4F2*, *CYP4A11* and other candidate genes played key roles in the pathogenesis of ischemic stroke [3, 10, 11]. *CYP1A1* and *CYP1A2* are the members of the CYP family participating in the metabolism of exogenous drugs, endogenous substrates, etc. Researchers [12, 13] reported that CYP1A1 and CYP1A2 played important roles in the bioactivation of polyunsaturated fatty acids, which can use NADPH to reduce the amount of O_2_, producing H_2_O_2_ and superoxide anion radicals [14]. Furthermore, induction of CYP1A1 can enhance oxidative stress response and increase the physiological production of ROS in skin [15, 16]. The above studies illustrated that CYP1A1 can induce oxidative stress induced by ROS overproduction. However, the exact mechanism of CYP1A1 and CYP1A2 in stroke remains unclear.

In this study, we used to explore the relationship between *CYP1A1* and *CYP1A2* genetic variants and the incidence of stroke in the Chinese population by the chi-square test and logistic-regression analysis, to provide theoretical basis for the function of CYP1A1 and CYP1A2 in stroke, and to provide more evidence for elucidating the cause of stroke.

## Methods

### Study design

Four hundred seventy-seven case samples and four hundred and eighty control samples were randomly collected from Traditional Chinese medicine of Baoji Hospital. All subjects were Shaanxi people of China. According to the World Health Organization’s diagnostic criteria for stroke, all cases were confirmed by professionals using computed tomography and/or magnetic resonance imaging. Cases with a history of cancer, inflammatory disease or other chronic diseases were excluded from this study. Every member of the control group had a rigorous physical examination to ensure that they were healthy without inflammatory disease and chronic diseases. Some additional information on the patients was also collected, including their history with hypertension, coronary heart disease, cerebral infarction and lumen infarction. Written informed consent was obtained from all subjects. The study period is from 2018 to 03 to 2019–12. Also, the study was approved by the Institutional Review Board of the Traditional Chinese medicine of Baoji Hospital (201802).

### SNP selection, genotyping and data collection

Totally, 4 variants (rs1048943, rs4646422, rs762551 and rs2470890) were selected in the global population on the basis of the 1000 Genomes Project (https://www.internationalgenome.org/) [17]. The minor allele frequency of every SNP was greater than 5%. Primers design for amplification and extension of SNPs listed in Supplementary Table 1 were completed by the Agena Bioscience Assay Design Suite V2.0 software (https://agenacx.com/online-tools/). Genomic DNA was extracted from the participants’ blood samples using the GoldMag-Mini Whole Blood Genomic DNA Purification Kit (GoldMag. Co. Ltd., Xi’an, China) regarded as the amplification template. Target amplification of SNPs was performed according to a predetermined PCR procedure, and PCR product purification was completed by agarose gel electrophoresis. After that, SNP genotyping was performed by the Agena MassARRAY platform with iPLEX gold chemistry (Agena Bioscience, San Diego, CA, USA), and data management was completed by Agena Bioscience TYPER, Version 4.0 [18].

**Fig. 1:**
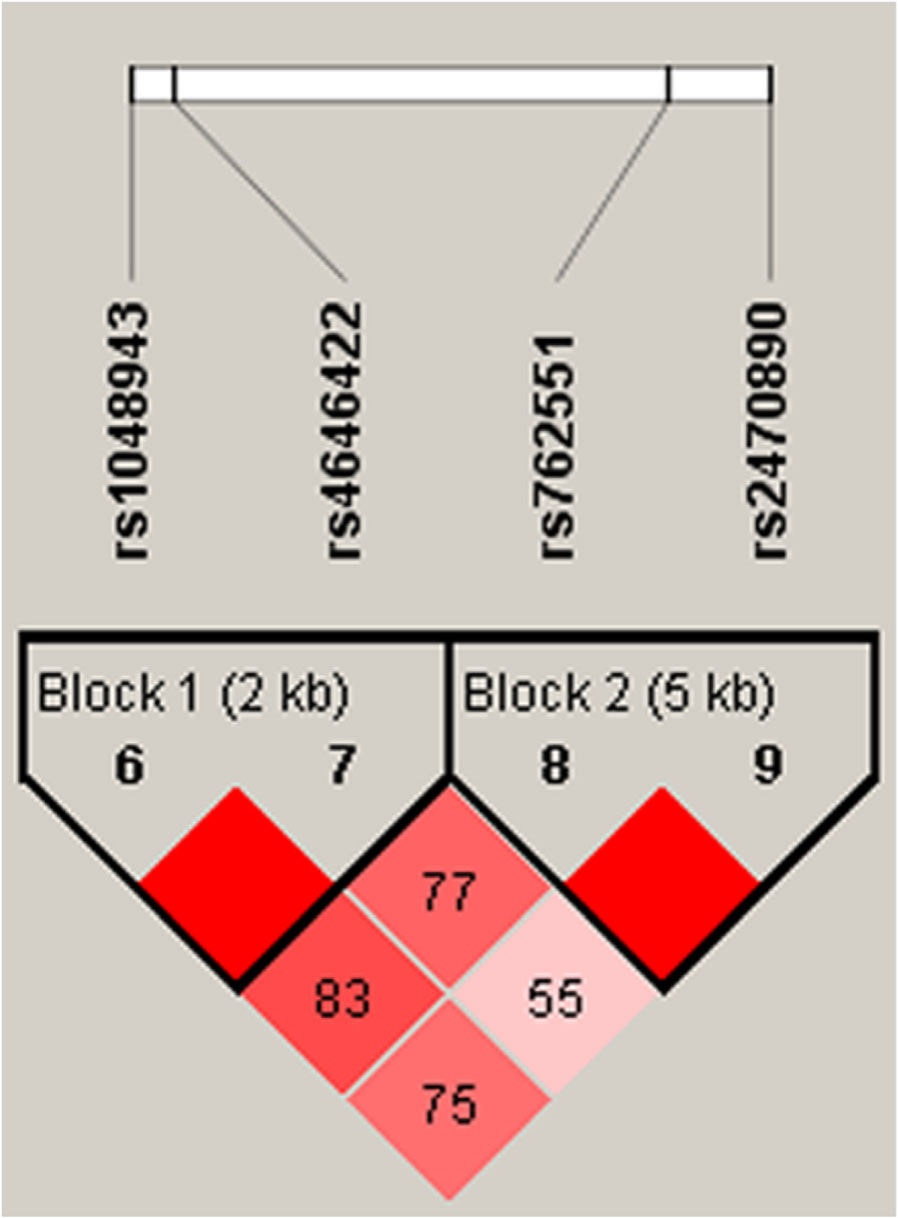
Linkage disequilibrium (LD) analysis of five SNPs in *CYP1A1*, and *CYP1A2*. The LD value is determined by r^2^ > 0.8 analyzed by Haploview software, version 4.2

### Statistical analysis

Based on the SPSS and Excel software, the allele frequencies for each SNP were calculated. Pearson’s chi-square test was used to assess whether the genotype distribution of variants among controls were in accordance with Hardy-Weinberg equilibrium. T-test and chi-square test were utilized to estimate the differences of age and gender distribution between cases and controls, respectively. Logistic regression analysis presented by the PLINK software, version 1.07 (Harvard, Boston, MA, USA) was used to estimate the association between *CYP1A1* and *CYP1A2* variants and stroke risk. Linkage disequilibrium (LD) using the Haploview software, version 4.2 (Harvard, Boston, MA, USA) was detected among the SNPs of *CYP1A1* and *CYP1A2* with HWE (*P* > 0.001). All *P*-values were two-tailed and *P*<0.05 were considered significant. Regardless of the level of significance found, all of the comparisons made will be considered exploratory in nature.

## Results

### Basic information of participants

In this study, 316 out of 477 cases were males and 161 were females, while the number of men and women among the 480 controls was 313 and 167, respectively. Background information on the participants was listed in Table 1. The mean age of cases and controls were 64.13 ± 10.82 years old and 63.69 ± 6.69 years old, respectively, indicating that there was no significant difference in age between cases and controls. Genotype frequency of each SNP in the controls was in accordance with HWE (*P* > 0.05) (Supplementary Table 2).

**Table 1:** Basic information of the participants

Characteristics	Cases *N*(%)	Controls *N*(%)	*P*-value
Number	477	480	
Gender, no, %			0.735^a^
Male	316 (66.2%)	313 (65.2%)	
Female	161 (33.8%)	167 (34.8%)	
Age, year (mean ± SD)	64.13 ± 10.82	63.69 ± 6.69	0.442^b^
> 64	229 (48.0%)	195 (40.6%)	
≤ 64	248 (51.7%)	285 (59.4%)	
Hypertension			
Yes	340 (71%)		
No	137 (29%)		
Coronary heart disease			
Yes	103 (22%)		
No	374 (78%)		
Cerebral infarction			
Yes	360 (75%)		
No	117 (25%)		
Lacunar infarction			
Yes	102 (21%)		
No	375 (79%)		

^a^ *P* values were calculated by two-sided Chi-square tests

^b^ *P* values were calculated by Student t-test

Bold-face values indicate statistical significance (*P* < 0.05)

### Overall analysis of the association between CYP1A1 and CYP1A2 variants and stroke risk

As shown in Table 2, allele C of *CYP1A2* rs762551 was associated with a lower risk of stroke than allele A (OR = 0.82, 95% CI: 0.68–0.99, *P* = 0.034). Overall analysis showed a link between the SNP and stroke risk (Table 2). Without adjustment, rs762551 was related to stroke risk in the codominant (*P* = 0.010), recessive (*P* = 0.005) and log-additive (*P* = 0.032) models (no shown). After adjusted for gender and age, the codominant model showed that AA carriers had a 0.60-times lower risk of stroke than CC carriers (OR = 0.60, 95% CI: 0.40–0.88, *P* = 0.010). The recessive model indicated that individuals with AA genotype had a lower risk of stroke compared to the C/C-A/C genotype carriers (OR = 0.60, 95% CI: 0.42–0.86, *P* = 0.005). The log-additive model also demonstrated that the SNP was in connection with a decreased risk of stroke (OR = 0.81, 95% CI: 0.68–0.98, *P* = 0.031).

**Table 2:** The association between polymorphisms of *CYP1A1* and *CYP1A2* and stroke risk

SNP-ID	Model	Genotype	Frequency		Without adjustment		With adjustment	
			Case	Control	OR (95% CI)	*P*^a^-value	OR (95% CI)	*P*^b^-value
rs1048943	codominant	C/C	28	27	1		1	
		C/T	167	185	0.86 (0.66–1.12)	0.262	0.86 (0.66–1.13)	0.284
		T/T	282	268	0.99 (0.57–1.72)	0.959	0.98 (0.56–1.71)	0.950
	dominant	C/C	28	27	1		1	
		C/T-T/T	449	453	0.87 (0.68–1.13)	0.304	0.88 (0.68–1.14)	*0.324*
	recessive	C/C-C/T	195	212	1		1	
		T/T	282	268	1.05 (0.61–1.80)	*0.871*	1.04 (0.60–1.80)	0.889
	log-additive	–	–	–	0.92 (0.75–1.14)	0.436	0.92 (0.75–1.138)	0.451
rs4646422	codominant	T/T	12	11	1		1	
		T/C	128	135	0.94 (0.70–1.25)	0.654	0.94 (0.70–1.25)	0.651
		C/C	336	332	1.08 (0.47–2.48)	0.860	1.07 (0.46–2.46)	0.876
	dominant	T/T	12	11	1		1	
		T/C-C/C	464	467	0.95 (0.72–1.25)	0.703	0.95 (0.72–1.25)	0.696
	recessive	T/T-T/C	140	146	1		1	
		C/C	336	332	1.10 (0.48–2.51)	0.825	1.09 (0.48–2.49)	0.840
	log-additive	–	–	–	0.97 (0.76–1.24)	0.785	0.96 (0.75–1.23)	0.775
rs762551	codominant	C/C	58	90	1		1	
		A/C	245	228	0.99 (0.75–1.32)	0.965	0.99 (0.75–1.31)	0.947
		A/A	173	160	0.60 (0.40–0.88)	0.010	0.60 (0.40–0.88)	**0.010**
	dominant	C/C	58	90	1		1	
		A/C-A/A	418	388	0.88 (0.68–1.15)	0.352	0.88 (0.67–1.15)	0.341
	recessive	C/C-A/C	303	318	1		1	
		A/A	173	160	0.60 (0.42–0.86)	0.005	0.60 (0.42–0.86)	**0.005**
	log-additive	–	–	–	0.81 (0.68–0.98)	0.032	0.81 (0.68–0.98)	**0.031**
rs2470890	codominant	T/T	8	6	1		1	
		T/C	102	101	1.03 (0.75–1.40)	0.856	1.02 (0.75–1.40)	0.883
		C/C	366	373	1.36 (0.47–3.96)	0.574	1.35 (0.46–3.95)	0.579
	dominant	T/T	8	6	1		1	
		T/C-C/C	468	474	1.05 (0.77–1.42)	**0.763**	1.04 (0.77–1.41)	0.791
	recessive	T/T-T/C	110	107	1		1	
		C/C	366	373	1.35 (0.47–3.92)	**0.581**	1.35 (0.46–3.92)	0.585
	log-additive	–	–	–	1.06 (0.81–1.40)	0.676	1.06 (0.80–1.39)	0.701

*SNP* Single nucleotide polymorphism, *OR* Odds ratio, *95% CI* 95% confidence interval

*P*^a^-values were calculated by logistic regression analysis withoutment

*P*^b^-values were calculated by logistic regression analysis adjusted for gender and age

^*^Bold-face values indicate statistical significance (*P* < 0.05)

### Stratification analysis by age and gender

To further investigate the correlation between variants of *CYP1A1*, *CYP1A2* and stroke risk, stratification analysis was also completed (Table 3). After age stratified by average age, the correlation between *CYP1A2* rs762551 and stroke risk was analyzed. The SNP was only found to be linked with a reduced risk of stroke in ≤64ys age group (OR = 0.56, 95% CI: 0.33–0.92, *P* = 0.024) in the recessive model, but, the association was not significant in >64ys age group. After gender stratification, there was a significant association between rs762551 and stroke risk in males, but not in females. The codominant model showed that rs762551 AA genotype decreased stroke risk by 0.56-fold (*P* = 0.017). However, no significant differences were found in other models.

**Table 3:** Stratified analysis of the association of *CYP1A2* polymorphism with stroke risk

SNP	Model	Genotype	>64ys		≤64ys		Males		Females	
			OR(95% CI)	*P*-values	OR(95% CI)	*P*-values	OR(95% CI)	*P*-values	OR(95% CI)	*P*-values
rs762551	codominant	C/C	1		1		1		1	
		A/C	0.75 (0.48–1.18)	0.216	1.33 (0.89–2.00)	0.166	1.04 (0.74–1.48)	0.815	0.90 (0.56–1.45)	0.666
		A/A	0.62 (0.33–1.18)	0.148	0.66 (0.38–1.16)	0.145	0.56 (0.34–0.90)	0.017^*^	0.68 (0.34–1.35)	0.267
	dominant	C/C	1		1		1		1	
		A/C-A/A	0.72 (0.47–1.11)	0.137	1.12 (0.76–1.65)	0.562	0.89 (0.64–1.24)	0.503	0.85 (0.54–1.34)	0.472
	recessive	C/C-A/C	1		1		1		1	
		A/A	0.74 (0.41–1.32)	0.302	0.56 (0.33–0.92)	0.024^*^	0.74 (0.41–1.32)	0.302	0.72 (0.39–1.35)	0.306
	log-additive	–	0.78 (0.58–1.06)	0.111	0.89 (0.69–1.16)	0.407	0.80 (0.64–1.00)	0.053	0.84 (0.61–1.16)	0.296

*SNP* Single nucleotide polymorphism, *OR* Odds ratio, *95% CI* 95% confidence interval, *ys* Years

*P*-values were calculated from logistic regression analysis adjusted for gender and/or age

^*^Bold-face values indicate statistical significance (*P* < 0.05)

### Analysis of the association between CYP1A1, CYP1A2 variants and stroke risk in patients with hypertension and coronary heart disease

The association between variants of *CYP1A1*, *CYP1A2* and stroke susceptibility was evaluated in patients with hypertension and coronary heart disease shown in Table 4. By comparing with non-hypertensive cases, the correlation between *CYP1A1* rs4646422, *CYP1A2* (rs762551 and rs2470890) and stroke risk in hypertensive cases was first analyzed in the allele model (Table 4). Allele C of rs2470890 conferred an increased susceptibility to stroke compared to the allele T (adjusted OR = 1.68, 95% CI: 1.05–2.69, *P* = 0.030). In the codominant model, compared with the carriers of TT genotype, subjects with TC genotype of *CYP1A1* rs4646422 had a negative effect on the risk of stroke (adjusted OR = 1.69, 95% CI: 1.04–2.74, *P* = 0.035). However, in the recessive model, CC genotype played a protective effect on the risk of stroke (adjusted OR = 0.28, 95% CI: 0.09–0.89, *P* = 0.032) compared to the T/T-T/C genotype. Additionally, there was a link between *CYP1A2* rs762551 and stroke susceptibility in the codominant (adjusted OR = 1.65, 95% CI: 1.08–2.54, *P* = 0.022) and dominant (adjusted OR = 1.57, 95% CI: 1.04–2.36, *P* = 0.030) models. Moreover, *CYP1A2* rs2470890 was associated with stroke risk in the codominant (adjusted OR = 1.91, 95% CI: 1.11–3.27, *P* = 0.019), dominant (adjusted OR = 1.85, 95% CI: 1.10–3.10, *P* = 0.021) and log-additive (adjusted OR = 1.67, 95% CI: 1.04–2.70, *P* = 0.035) models.

**Table 4:** The association between *CYP1A1* and *CYP1A2* polymorphisms and stroke risk in patients with hypertension and coronary heart disease

	SNP	Model	Genotype	Case	Control	Adjustment for age and gender	
						OR(95% CI)	*P*-values
Hypertension							
*CYP1A1*	rs4646422	Allele	T	111	41	1	
			C	567	233	1.11 (0.75–1.64)	0.591
		codominant	T/T	5	7	1	
			T/C	101	27	1.69 (1.04–2.74)	0.035^*^
			C/C	233	103	0.32 (0.10–1.03)	0.055
		dominant	T/T	5	7	1	
			T/C-C/C	334	130	1.40 (0.89–2.21)	0.142
		recessive	T/T-T/C	106	34	1	
			C/C	233	103	0.28 (0.09–0.89)	0.032^*^
		log-additive	–	–	–	1.13 (0.76–1.67)	0.546
*CYP1A2*	rs762551	Allele	C	267	94	1	
			A	411	180	1.24 (0.93–1.67)	0.144
		codominant	C/C	41	17	1	
			A/C	185	60	1.65 (1.08–2.54)	0.022^*^
			A/A	113	60	1.28 (0.67–2.46)	0.451
		dominant	C/C	41	17	1	
			A/C-A/A	298	120	1.57 (1.04–2.36)	0.030^*^
		recessive	C/C-A/C	226	77	1	
			A/A	113	60	0.97 (0.53–1.78)	0.918
		log-additive	–	–	–	1.28 (0.94–1.74)	0.122
	rs2470890	Allele	T	94	24	1	
			C	584	250	1.68 (1.05–2.69)	0.030^*^
		codominant	T/T	6	2	1	
			T/C	82	20	1.91 (1.11–3.27)	0.019^*^
			C/C	251	115	1.22 (0.24–6.22)	0.809
		dominant	T/T	6	2	1	
			T/C-C/C	333	135	1.85 (1.10–3.10)	0.021^*^
		recessive	T/T-T/C	88	22	1	
			C/C	251	115	1.08 (0.21–5.47)	0.928
		log-additive	–	–	–	1.67 (1.04–2.70)	0.035^*^
Coronary heart disease							
*CYP1A1*	rs4646422	Allele	T	43	109	1	
			C	163	637	1.54 (1.04–2.28)	0.030^*^
		codominant	T/T	3	9	1	
			T/C	37	91	1.79 (1.11–2.90)	0.017^*^
			C/C	63	273	1.32 (0.34–5.12)	0.690
		dominant	T/T	3	9	1	
			T/C-C/C	100	364	1.75 (1.10–2.78)	0.019^*^
		recessive	T/T-T/C	40	100	1	
			C/C	63	273	1.10 (0.29–4.24)	0.886
		log-additive	–	–	–	1.54 (1.03–2.30)	0.036^*^
*CYP1A2*	rs2470890	Allele	T	18	100	1	
			C	188	646	0.62 (0.37–1.05)	0.072
		codominant	T/T	1	7	1	
			T/C	16	86	0.57 (0.31–1.03)	0.060
			C/C	86	280	0.43 (0.05–3.67)	0.444
		dominant	T/T	1	7	1	
			T/C-C/C	102	366	0.56 (0.31–0.99)	0.047^*^
		recessive	T/T-T/C	17	93	1	
			C/C	86	280	0.49 (0.06–4.10)	0.508
		log-additive	–	–	–	0.58 (0.34–1.00)	0.049

*OR* Odds ratio, *95% CIs* 95% confidence intervals

*P*-values were calculated by logistic regression analysis adjusted for gender and age

^*^Bold-face values indicate statistical significance (*P* < 0.05)

In patients with coronary heart disease (Table 4), individuals with rs4646422 C allele were found to be increased the likelihood of stroke by 1.54 times when compared with the T allele (*P* = 0.030). In addition, the variant was related to an increased risk of stroke (codominant: adjusted OR = 1.79, 95% CI: 1.11–2.90, *P* = 0.017; dominant: adjusted OR = 1.75, 95% CI: 1.10–2.78, *P* = 0.019; log-additive: adjusted OR = 1.54, 95% CI: 1.03–2.30, *P* = 0.036). Rs2470890 was correlated with a decreased risk of stroke in the recessive model (adjusted OR = 0.56, 95% CI: 0.31–0.99, *P* = 0.047).

### Analysis of the association between CYP1A1, CYP1A2 variants and stroke risk in patients with cerebral infarction and lacunar infarction

In addition, we analyzed the association between *CYP1A1* rs1048943 and stroke risk in cerebral infarction cases vs non-cerebral infarction cases (Supplementary Table 3). The recessive model showed that subjects with genotype TT were more likely to suffer from stroke than those with C/C-C/T genotypes before adjustment (OR = 4.48, 95% CI: 1.05–19.15, *P* = 0.043). Moreover, the relationship between *CYP1A1* rs1048943 and stroke risk was analyzed in lacunar infarction cases vs non-lacunar infarction cases adjusted for age and gender (Supplementary Table 4). The variant was related to a decreased risk of stroke in the codominant (OR = 0.13, 95% CI: 0.02–0.96, *P* = 0.045) and recessive (OR = 0.13, 95% CI: 0.02–0.95, *P* = 0.045) models.

### LD and haplotype analysis

Among the four SNPs (rs1048943, rs4646422, rs762551 and rs2470890), LD analysis was performed. A strong linkage between the SNPs was observed (Fig. 1). Table 5 listed all of the possible haplotypes of the rs762551 and rs2470890. In the overall analysis results, the frequencies of the most dominant haplotype AT was 0.876 and 0.882 in cases and controls, respectively. The CC haplotype was the disadvantaged haplotype with frequencies of 0.380 and 0.427 in cases and controls, respectively. Besides, CC haplotype was still correlated with a reduced risk of stroke (OR = 0.82, 95% CI: 0.68–0.99, *P* = 0.035; adjusted OR = 0.82, 95% CI: 0.68–0.99, *P* = 0.034). In addition, clinical characteristics analysis in patients with hypertension indicated that AT haplotype was associated with a decreased susceptibility of stroke (OR = 0.60, 95% CI: 0.37–0.96, *P* = 0.032; adjusted OR = 0.60, 95% CI: 0.37–0.96, *P* = 0.033). AC haplotype was associated with an increased susceptibility of stroke (OR = 1.56, 95% CI: 1.16–2.10, *P* = 0.003; adjusted OR = 1.57, 95% CI: 1.16–2.11, *P* = 0.003).

**Table 5:** Haplotype frequencies of *CYP1A2* SNPs and the association with stroke risk

	SNP	Haplotype	Frequency		Without adjustment		With adjustment	
			Case	Control	OR(95% CI)	*P*-values	OR(95% CI)	*P*-values
Total	rs762551|rs2470890	AT	0.876	0.882	0.95 (0.72–1.25)	0.688	0.95 (0.72–1.25)	0.718
	rs762551|rs2470890	CC	0.380	0.427	0.82 (0.68–0.99)	0.035^*^	0.82 (0.68–0.99)	0.034^*^
	rs762551|rs2470890	AC	0.504	0.545	0.85 (0.71–1.02)	0.076	0.85 (0.71–1.01)	0.070
Hypertension	rs762551|rs2470890	AT	0.861	0.912	0.60 (0.37–0.96)	0.032^*^	0.60 (0.37–0.96)	0.033^*^
	rs762551|rs2470890	CC	0.395	0.343	1.28 (0.94–1.74)	0.117	1.29 (0.94–1.75)	0.113
	rs762551|rs2470890	AC	0.534	0.431	1.56 (1.16–2.10)	0.003^*^	1.57 (1.16–2.11)	0.003^*^

*OR* Odds ratio, *95% CIs* 95% confidence intervals

*P*-values were calculated by logistic regression with adjustment for age and gender

^*^Bold-face values indicated statistical significance (*P* < 0.05)

## Discussion

This study investigated the association between *CYP1A1* variants and *CYP1A2* and stroke risk in the Chinese Han population. Except for rs1048943, this is the first study to show that rs4646422, rs762551 and rs2470890 were associated with stroke risk. The four SNPs were found to be correlated with stroke risk in patients with hypertension, coronary heart disease, cerebral infarction and lacunar infarction.

Recently, researches have also shown that *CYP1A1* variants played a role in the development of ischemic stroke (IS) susceptibility in different populations, such as Turk [19], Indian [20] and Chinese [21]. Demirdöğen et al. [19] reported that rs1048943 and rs4646903 may play a significant role in smoking- and hypertension-induced IS risk in the Turkish population. Among the 6235C carriers or 4889G carriers, the prevalence of hypertension and IS risk associated with hypertension were lower than that of wild type carriers. Sultana et al. [20] observed that South Indian population with *CYP1A1* rs4646903 CC genotype was related to an increased risk of IS, up to 5.14 times, while the other genotypes had no influence on the IS risk. Additionally, Zhang et al. [21] revealed that rs4646903 and rs1048943 of *CYP1A1* were related to IS risk in eastern China. This illustrated that individuals with TC-CC genotype of rs4646903 and AG-GG genotype of rs1048943 had a lower IS risk compared to rs4646903 TT and rs1048943 AA, respectively. Whereas, rs4646922 was not found to be associated with IS risk. In this study, rs4646922 was associated with stroke risk in the Shaanxi population of China. Given the inconsistency in the above results, ethnic differences and population distribution may be an important reason.

Moreover, Mega et al. analyzed the effect of Prasugrel on CYP gene polymorphisms and found non-significant relationship between *CYP1A2* SNPs and stroke risk [4]. Until now, the correlation between *CYP1A2* variants and stroke risk has not been reported. In this study, the results first illustrated that rs4646422, rs762551 and rs2470890 were found to be related to stroke risk in Shaanxi population of China. In future, it needs to be tested in a larger sample to provide evidence for stroke risk prediction.

### Study strengths and limitations

The study has several strengths. Firstly, the strength is the genotype distribution of variants among controls in accordance with HWE *P*-value, which may reduce bias to some extent. Secondly, although the number of cases and controls was not enough, it was enough for statistical analysis, and the results were adjusted by age and gender to eliminate data defects.

Moreover, this study has several limitations. First, insufficient sample size may affect the research conclusion. Then the larger sample size is needed to verify the results. Second, the sample is limited to one race, which may lead to some uncertainty in the results of the study. Later, data from different ethnic groups is needed to verify the results. Third, the study is not thorough due to a lack of information on smoking, drinking and body mass index. Finally, the results only showed that polymorphisms of *CYP1A1* and *CYP1A2* were related to the risk of stroke, and there was no more clear mechanism study. RegulomeDB (https://www.regulomedb.org/regulome-search/) and HaploReg v4.1 (https://pubs.broadinstitute.org/mammals/haploreg/haploreg.php) are used to predict the effect of *CYP1A1* and *CYP1A2* polymorphisms on their function (Supplementary Table 5). Then, molecular experiment is used to further verify the role of *CYP1A1* and *CYP1A2* polymorphisms in the course of stroke.

## Conclusions

This was the first time that rs4646422, rs762551 and rs2470890 were observed to be related to stroke risk in the Chinese population, which is helpful for obtaining a more conclusive understanding of the function of CYP1A1 and CYP1A2 and for laying a foundation for further elucidating the pathogenesis of stroke.

## Supplementary information

**Additional file 1: Table S1.** Primers used for this study. **Table S2.** Basic information of candidate SNPs in *CYP1A1* and *CYP1A2*. **Table S3.** The association between *CYP1A1* polymorphism and stroke risk in patients with cerebral infarction. **Table S4.** The association between *CYP1A1* polymorphism and stroke risk in patients with lacunar infarction. **Fig. 1** Linkage disequilibrium (LD) analysis of five SNPs in *CYP1A1*, and *CYP1A2.* The LD value is determined by r^2^ > 0.8 analyzed by Haploview software, version 4.2. **Table S5**. In silico analysis for SNPs function annotation.

## Data Availability

The datasets generated and/or analyzed during the current study are not publicly available, but are available from the corresponding author on reasonable request.

## Abbreviations

SNPs: Single nucleotide polymorphisms
OR: Odds ratio
95% CI: 95% confidence interval
CYP1A: Cytochrome P450, family 1, subfamily A, polypeptide 1
LD: Linkage disequilibrium
HWE: Hardy-Weinberg Equilibrium
MAF: Minor allele frequency
IS: Ischemic stroke
