# Supplementary material for: The influence of CYP1A1 and CYP1A2 polymorphisms on stroke risk in the Chinese population
Source: Lipids Health Dis. 2020 Oct 12;19:221. doi: 10.1186/s12944-020-01370-z (PMC7552501; doi:10.1186/s12944-020-01370-z)

Supplementary Table 1. Primers used for this study.

| SNP_ID | 1st-PCR primer | 2nd-PCR primer | UEP SEQ | Direction |
| --- | --- | --- | --- | --- |
| rs1048943 | ACGTTGGATGTGGGCAAGCGGAAGTGTATC | ACGTTGGATGCTGAATTCCACCCGTTGCAG | ccctcCCTCCCAGCGGGCAA | F |
| rs4646422 | ACGTTGGATGTGGGTAATCAGGGCCTCAAG | ACGTTGGATGTCAGCATGTGCCCAATCAGA | gggtCCCAATCAGAGGCCAG | F |
| rs762551 | ACGTTGGATGGAATCTTGAGGCTCCTTTCC | ACGTTGGATGCTAAGCTCCATCTACCATGC | CTACCATGCGTCCTG | R |
| rs2470890 | ACGTTGGATGGCCTCAGAATGGTGGTGTCT | ACGTTGGATGTCTACGGGCTGACCATGAAG | CTGCGCTTCTCCATCAA | F |
| SNP, single nucleotide polymorphism; UEP SEQ, Unextended mini-sequencing primer. | | | | |

Supplementary Table 2 Basic information of candidate SNPs in *CYP1A1* and *CYP1A2*.

| SNP-ID | Chr | Gene | Position | Alleles | MAF | | HWE-  *P*-values |
| --- | --- | --- | --- | --- | --- | --- | --- |
|  |  |  |  | (minor/major) | Cases | Controls |  |
| rs1048943 | 15 | *CYP1A1* | 74720644 | C/T | 0.234 | 0.249 | 0.544 |
| rs4646422 | 15 | *CYP1A1* | 74722964 | T/C | 0.160 | 0.164 | 0.619 |
| rs762551 | 15 | *CYP1A2* | 74749576 | C/A | 0.379 | 0.427 | 0.576 |
| rs2470890 | 15 | *CYP1A2* | 74755085 | T/C | 0.124 | 0.118 | 1.000 |

SNP: single nucleotide polymorphism, MAF: minor allele frequency, HWE: Hardy-Weinberg equilibrium, OR: odds ratio, 95% CI: 95% confidence interval.

*P*-values were calculated from two-sided χ^2^ test.

^*^Bold-face values indicate statistical significance (*P* < 0.05).

Supplementary Table 3 The association between *CYP1A1* polymorphism and stroke risk in patients with cerebral infarction.

| SNP | Model | Genotype | Case | Control | Without adjustment | |
| --- | --- | --- | --- | --- | --- | --- |
|  |  |  |  |  | OR(95% CI) | *P*-values |
| rs1048943 | codominant | C/C | 26 | 2 | 1 |  |
|  |  | C/T | 121 | 46 | 0.85(0.55-1.32) | 0.471 |
|  |  | T/T | 213 | 69 | 4.21(0.97-18.20) | 0.054 |
|  | dominant | C/C | 26 | 2 | 1 |  |
|  |  | C/T-T/T | 334 | 115 | 0.99(0.65-1.52) | 0.971 |
|  | recessive | C/C-C/T | 147 | 48 | 1 |  |
|  |  | T/T | 213 | 69 | 4.48(1.05-19.15) | **0.043^*^** |
|  | log-additive | – | – | – | 1.16(0.82-1.65) | 0.409 |

OR: odds ratio; 95% CIs: 95% confidence intervals.

*P*-values were calculated from chi-square test.

^*^Bold-face values indicate statistical significance (*P* < 0.05).

Supplementary Table 4 The association between *CYP1A1* polymorphism and stroke risk in patients with lacunar infarction.

| SNP | Model | Genotype | Case | Control | With adjustment | |
| --- | --- | --- | --- | --- | --- | --- |
|  |  |  |  |  | OR(95% CI) | *P*-values |
| rs1048943 | codominant | C/C | 1 | 27 | 1 |  |
|  |  | C/T | 37 | 130 | 0.99(0.63-1.58) | 0.982 |
|  |  | T/T | 64 | 218 | 0.13(0.02-0.96) | **0.045^*^** |
|  | dominant | C/C | 1 | 27 | 1 |  |
|  |  | C/T-T/T | 101 | 348 | 0.84(0.54-1.32) | 0.457 |
|  | recessive | C/C-C/T | 38 | 157 | 1 |  |
|  |  | T/T | 64 | 218 | 0.13(0.02-0.95) | **0.045^*^** |
|  | log-additive | – | – | – | 0.74(0.51-1.09) | 0.131 |

OR: odds ratio; 95% CIs: 95% confidence intervals.

*P*-values were calculated from chi-square test.

^*^Bold-face values indicate statistical significance (*P* < 0.05).

Supplementary Table 5 In silico analysis for SNPs function annotation

| SNP | Chr | Gene | Allele | RegulomeDB Score | HaploReg |
| --- | --- | --- | --- | --- | --- |
| rs1048943 | 15 | *CYP1A1* | C/T | 3a | DNAse |
| rs4646422 | 15 | *CYP1A1* | T/C | 5 | Motifs changed |
| rs762551 | 15 | *CYP1A2* | C/A | 5 | Motifs changed, Selected eQTL hits |
| rs2470890 | 15 | *CYP1A2* | T/C | 1f | DNAse, Selected eQTL hits |

1f indicates that the variant is likely to affect binding and linked to expression of a gene target.

3a indicates that the variant is less likely to affect binding.

5 indicates that the variant has minimal binding evidence.

Figure 1 Linkage disequilibrium (LD) analysis of five SNPs in *CYP1A1*, and *CYP1A2.* The LD value is determined by r^2^ > 0.8 analyzed by Haploview software, version 4.2.


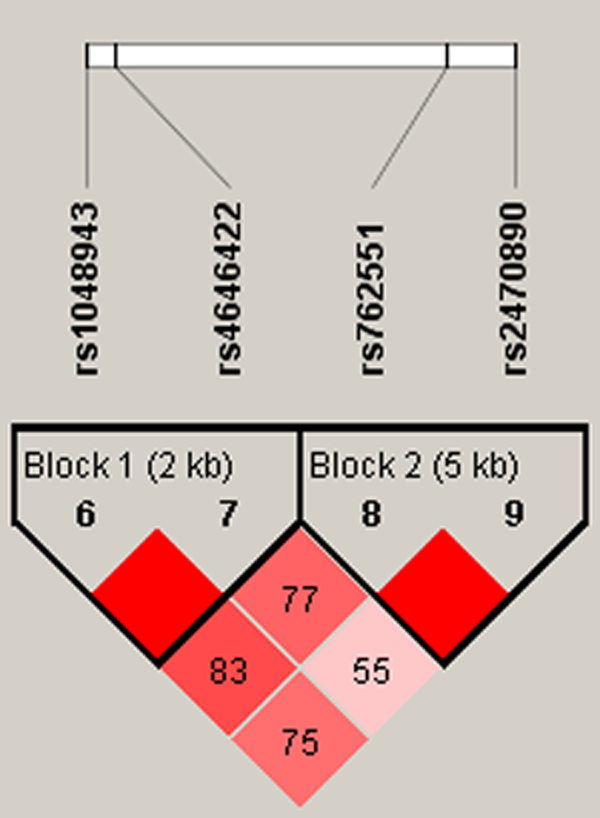

Supplement: Supplementary file 1 — Additional file 1: Table S1. Primers used for this study. Table S2. Basic information of candidate SNPs in CYP1A1 and CYP1A2. Table S3. The association between CYP1A1 polymorphism and stroke risk in patients with cerebral infarction. Table S4. The association between CYP1A1 polymorphism and stroke risk in patients with lacunar infarction. Fig. 1 Linkage disequilibrium (LD) analysis of five SNPs in CYP1A1, and CYP1A2. The LD value is determined by r2 > 0.8 analyzed by Haploview software, version 4.2. Table S5. In silico analysis for SNPs function annotation. [file 12944_2020_1370_MOESM1_ESM.docx]
